# First Reported Case of Successful Conception and Delivery During Stage IV Breast Cancer Treatment: A Case Report and Literature Review

**DOI:** 10.7759/cureus.47201

**Published:** 2023-10-17

**Authors:** Humaid O Al-shamsi, Nadia Abdelwahed, Mandeep Singh, Amin M Abyad, Shimaa Elsabae, Tamer Abdelgawad, Faryal Iqbal, Nuhad Ibrahim

**Affiliations:** 1 Department of Oncology, Burjeel Holdings Oncology Services, Burjeel Medical City, Abu Dhabi, ARE; 2 Department of Clinical Sciences, College of Medicine, Gulf Medical University, Ajman, ARE; 3 Emirates Oncology Society, Emirates Medical Association, Dubai, ARE; 4 Department of Medical Oncology, Burjeel Medical City, Abu Dhabi, ARE; 5 Department of Fetal Medicine and Therapy, Kypros Nicolaides Fetal Medicine and Therapy Center, Burjeel Medical City, Abu Dhabi, ARE; 6 Department of Radiology, Burjeel Medical City, Abu Dhabi, ARE; 7 Department of Research and Development, Burjeel Medical City, Abu Dhabi, ARE; 8 Department of Breast Medical Oncology, MD Anderson Cancer Center, Houston, USA

**Keywords:** pertuzumab, trastuzumab, malignancy, her2, pregnancy, breast cancer

## Abstract

We herein report a case involving a woman with metastatic human epidermal growth factor receptor 2 (HER-2)-positive breast cancer (BC) who became pregnant while undergoing active anticancer therapy with Trastuzumab-Pertuzumab for her advanced BC disease at our institution. To our knowledge, this is the first reported case of pregnancy and successful delivery in a stage IV BC patient during anticancer therapy. A multidisciplinary approach for such a complex case is a must to evaluate the mother’s medical condition by an experienced oncology team along with a maternal-fetal team, with support from a psychiatric and psychological evaluation for the mother. The use of effective contraception during anticancer therapy is essential to avoid such a scenario.

## Introduction

Breast cancer (BC) represents the most prevalent malignancy affecting the female population, assuming even greater significance as the leading cause of cancer-related mortality in women under the age of 40. Within the spectrum of BC, a distinct subtype is recognized as pregnancy-associated BC. This variant manifests during pregnancy, within the initial year postpartum, or during the lactation period. The incidence of pregnancy-associated BC has been observed to rise, mirroring the trend of delayed pregnancies in women. Pregnancy-associated BC is seen in 3 out of 1,000 pregnancies [1]. This particular BC subtype exhibits a heightened degree of aggressiveness, frequently compounded by diagnostic delays attributable to physiological alterations occurring during pregnancy and a discernible lack of awareness within the medical community.

While the diagnosis of advanced-stage cancer during pregnancy has been reported previously [2], to the best of our knowledge and based on our literature review as of October 2, 2023, there have been no previous reports of conception occurring while on active anticancer therapy for stage IV cancer.

## Case presentation

A 34-year-old premenopausal woman was diagnosed with clinically stage III invasive ductal carcinoma of the right breast (estrogen receptor [ER]-negative, progesterone receptor [PR]-negative, and human epidermal growth factor receptor 2 (HER-2)-positive +3 by immunohistochemistry) in September 2015. The patient traveled to the MD Anderson Cancer Center, Houston, TX. She underwent upfront right mastectomy and axillary lymph node dissection with reconstruction, followed by adjuvant chemotherapy with dose-dense adjuvant doxorubicin and cyclophosphamide. Subsequently, she received Trastuzumab and paclitaxel for four cycles, followed by Trastuzumab as a single agent (Pertuzumab was not approved at that time). Unfortunately, her treatment course was complicated with cardiomyopathy. In early June 2016, the patient developed symptomatic cardiomyopathy with an ejection fraction (EF) of 30% to 40%. Consequently, Trastuzumab was discontinued. Echocardiography (ECHO) was performed monthly to monitor cardiac function, and this remained stable, but no improvement in the EF was noted. Trastuzumab was kept on hold for six months. She had reconstructive surgery in March 2017. Subsequently, her EF improved by over 55%, and she then received two more cycles of Trastuzumab, with the last cycle in August 2017 (a total of 11 cycles). In October 2017, follow-up scans showed recurrent disease in the lung and mediastinal lymph nodes, with a biopsy from the lung proven to be metastases from her primary BC with ER-/PR-negative and HER-2-positive +3 by immunohistochemistry as the primary tumor at the time of diagnosis. Genetic profiling was negative for driver mutations (ATM, BRCA1, BRCA2, CDH1, CHEK2, PALB2, PTEN, and TP53). Given the aforementioned findings, her treatment was changed to the second line with ado-Trastuzumab Emtansine (TDM-1). She received 12 cycles with good response but was discontinued due to debilitating fatigue and grade 4 liver toxicity. The patient had minimal active disease on positron emission tomography-computed tomography (PET-CT) restaging imaging. She was then switched to dual-antibody Trastuzumab-Pertuzumab (Pertuzumab was introduced for the first time to the patient at this point) starting in February 2018, with no evidence of disease on follow-up scans and interruption of treatment multiple times due to a drop in EF <40%. The patient kept closely following cardiology. She discontinued all treatment starting in January 2020 due to another decline in EF, which decreased to 40%. After the recovery of EF >50%, the patient elected to resume Trastuzumab-Pertuzumab with close monitoring. Her evaluation with a CT scan in April 2020 showed no evidence of disease. The patient continued to be on Trastuzumab-Pertuzumab since then, but she was not compliant with treatment schedules on several occasions due to personal and social circumstances. Her last imaging was done in September 2022, with no evidence of disease.

The patient maintained an intrauterine device (IUD) throughout her treatment, with regular exchanges as advised by her gynecologist. Subsequently, she chose to remove the IUD to become pregnant, a decision made without consulting her oncology medical team.

In December 2022, after 12 days of her last cycle of treatment, she presented to the clinic for an emergency consultation, as she had been found through a home-based pregnancy test to be pregnant for at least 10 weeks based on her last menstrual period. The patient was counseled and advised to consider termination of pregnancy since she has been recently exposed to dual blockage with anti-HER-2 agents (Trastuzumab-Pertuzumab). The patient was committed to pregnancy and was aware that we would not be able to give any treatment for the remainder of the seven to eight months of pregnancy and that this might impact her prognosis at this stage of her disease. She was also fully aware of the risks of fetal malformations and the possible poor outcome that was likely to impact her and the fetus. The patient was also referred to a clinical psychologist for a full assessment, but the patient declined. After a full and informed decision, the patient was very clear about her wish to continue the pregnancy despite the risk of progression of her BC while she was off treatment for an extended period. The decision was thus made to discontinue all treatment and monitor her until delivery. She was referred to the maternal and fetal medicine clinic, where she was monitored closely.

At the Maternal Fetal Medicine Clinic, we performed a detailed 11- to 14-week scan, including a cell-free fetal DNA test, to exclude common chromosomal abnormalities. Counseling regarding the risk of structural malformations and chromosomal abnormalities was provided, and we quoted an overall risk of 1% to 5% for the same. A baseline pulmonary function test, electrocardiography, and ECHO were performed. The patient was fully committed to pregnancy despite acknowledging the associated risks, which included the potential for deterioration in cardiac function and the recurrence of BC. A further scan at 16 weeks and an anomaly scan at 20 weeks were performed. Uterine artery Doppler at 20 weeks was within the normal range, indicating a low risk for preeclampsia and/or fetal growth restriction. Anomaly scans and fetal ECHO did not detect any major or minor congenital malformation. Serial growth scans were planned for 26 weeks’ gestation, and the fetal growth was found to be consistent with the gestational age, exhibiting normal liquor volume and fetal Doppler results. A repeat pulmonary function test and ECHO were performed at 32 weeks gestation, and these were reported to be stable. She had four previous vaginal deliveries, and hence, we anticipated a spontaneous vaginal delivery. At 38 weeks gestation, she had a spontaneous rupture of membranes and went into labor after six hours, which culminated in a spontaneous vaginal delivery with a healthy male child. The newborn's APGAR score was 9 at 5 minutes and 10 at 10 minutes, indicating normal well-being.

A list of recommended tests for monitoring during pregnancy is listed in Table 1 [3].

**Table 1:** Recommended cardiac tests for monitoring during pregnancy. Source: [3].

Diagnostic test	Significance
Complete blood count	To evaluate anemia exclude infection
Beta-type natriuretic peptide (BNP)	To confirm diagnosis of heart failure when the patient develops increasing symptoms during pregnancy
Electrocardiography (ECG)	To provide information related to ventricular hypertrophy, myocardial ischemic disease, or arrhythmia
Echocardiography	To evaluate structural causes of heart failure such as valvular heart disease and provide information about cardiac dimensions, wall motion, and left ventricular ejection fraction
Chest X-ray	To evaluate cardiomegaly and pulmonary edema
Thyroid function test	To exclude hypothyroidism and hyperthyroidism

**Figure 1:**
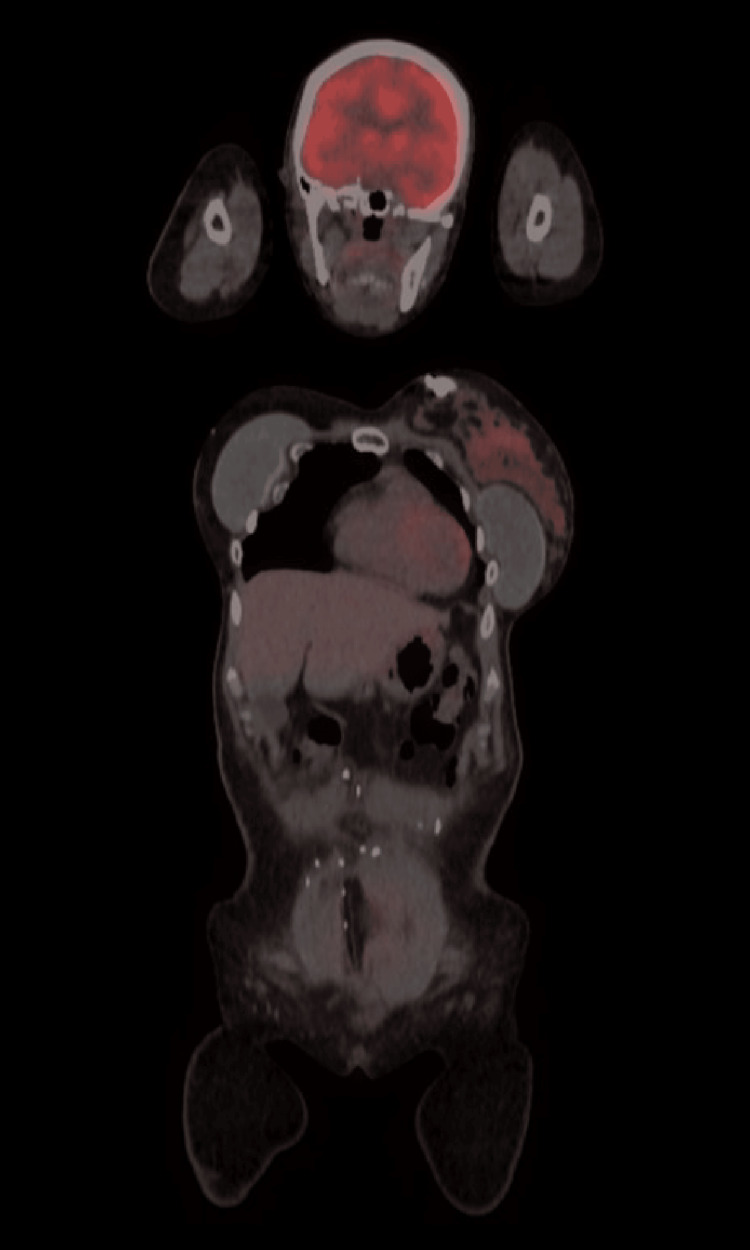
Sagittal PET-CT view of the case status postpartum with no evidence of disease and only physiological uptake findings in the left breast with no metastasis. PET-CT, positron emission tomography-computed tomography

**Figure 2:**
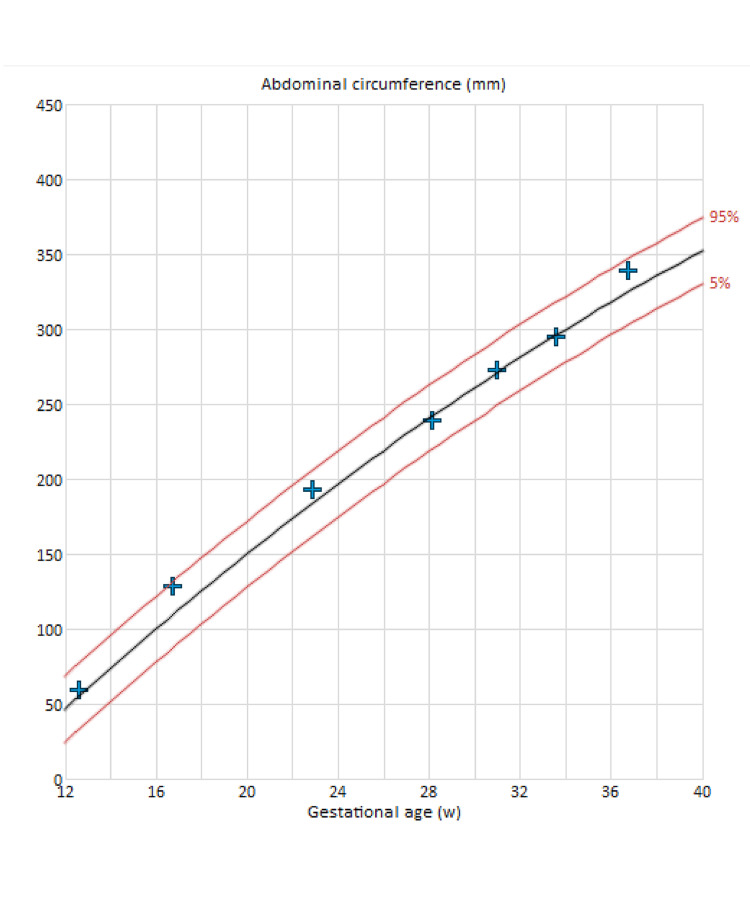
The abdominal circumference, assessed throughout the gestational age, indicates that the baby is growing on the 50th percentile, displaying normal growth.

**Figure 3:**
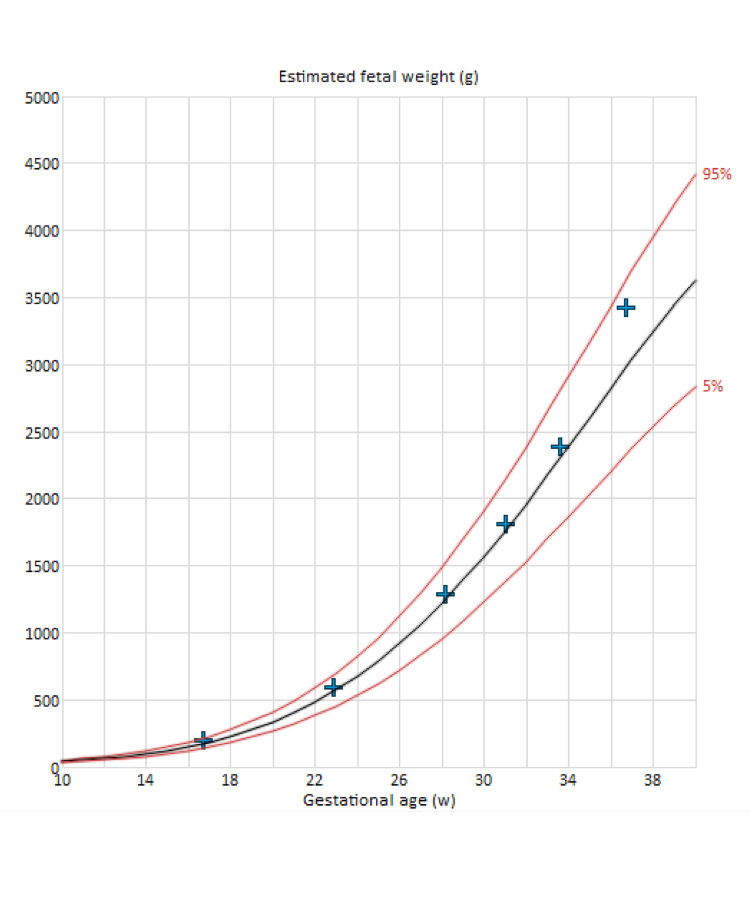
The estimated fetal weight throughout the gestational age indicates that the baby is growing on the 50th percentile, demonstrating normal growth.

She presented to our oncology clinic three weeks postpartum for an assessment, and her PET-CT was negative for any abnormal findings or metastatic disease (physiological findings in the left breast with no metastasis) (Figure 1). ECHO was also conducted with a normal EF >55%. A referral for a specialized neonatal cardiac assessment was completed with a normal evaluation of the infant (Figures 2-3).

The patient was counseled about the resumption of her therapy with either Trastuzumab-Pertuzumab or single-agent Trastuzumab. The patient chose to wait for three months before considering further action, as she expressed a desire to breastfeed her child (Table 2).

**Table 2:** Chronological sequence of events. ^*^Pertuzumab was approved in the United States in December 2017.

Year	Milestone
September 2015	A new diagnosis of breast cancer at the age of 34, and systemic therapy started with chemotherapy and Trastuzumab
June 2016	Cardiomyopathy and decline in ejection fraction due to Trastuzumab toxicity proven to be reversible treatment held for six months
August 2017	Completion of adjuvant Trastuzumab (a total of 11 cycles) with multiple interruptions
October 2017	Recurrent disease in the lung and mediastinal lymph nodes led to the initiation of ado-Trastuzumab Emtansine (TDM-1) for 12 cycles
January 2018	Completion of TDM-1 for 12 cycles with severe side effects, resulting in stopping the treatment
February 2018	Introduction of Trastuzumab and Pertuzumab^*^
January 2020	No evidence of disease; treatment stopped due to a decline in the ejection fraction
April 2020	Trastuzumab and Pertuzumab resumed after the recovery of the ejection fraction
October 2022	Elective removal of intrauterine device (IUD) without counseling the oncology team
December 2022	The initial presentation revealed a positive home pregnancy test indicating a 10-week pregnancy. Counseling regarding abortion was discussed, but the patient declined. Antenatal care was subsequently initiated.
August 2023	Delivery of a healthy baby at 38 weeks’ gestation due to the spontaneous rupture of the membrane
September 2023	The follow-up PET scan revealed no evidence of disease, and the patient expressed a desire to wait three months before resuming her Trastuzumab and Pertuzumab therapy to breastfeed.

## Discussion

BC is widely recognized as the most prevalent form of cancer worldwide, including in the UAE [4]. In the UAE, BC commonly occurs in women younger than 50 years, with 25% of invasive types harboring HER-2 overexpression [4]. It is estimated that 1 in 1,000 pregnant women may develop BC, which is the most common type of cancer occurring during pregnancy [5]. Most reported cases have locally advanced disease with large tumor sizes and involvement of axillary lymph nodes [6], with around 30% being HER-2-positive [6].

Pregnancy while undergoing active systemic therapy for BC is a complex and delicate situation. Pregnancy during stage IV cancer is a rare occurrence, and to our knowledge, the possibility and outcomes of pregnancy during active systemic cancer therapy in young women with metastatic cancers are poor, and the only reported case of BC stage IV we identified in our literature review led to the death of both mother and fetus [7].

In our case, the patient got pregnant while she was on active therapy with Trastuzumab and Pertuzumab. Treatment with Trastuzumab during pregnancy can lead to severe complications for both the mother and the baby [8], with oligohydramnios/anhydramnios being the most prevalent complication (61.1%) [8]. A large systematic review and meta-analysis of 17 studies (18 pregnancies; 19 newborns) assessing the use of Trastuzumab in treating BC during pregnancy reported that at the long-term evaluation, all children without problems at birth were healthy, with a median follow-up of nine months, while four out of nine children facing troubles at birth were dead within an interval ranging between birth and 5.25 months. All children exposed to Trastuzumab in utero exclusively in the first trimester were completely healthy at birth. The study concluded that Trastuzumab should not be administered during pregnancy. However, for women who become accidentally pregnant during Trastuzumab administration and wish to continue pregnancy, Trastuzumab should be stopped and pregnancy could be allowed to continue [8].

Another more recent, larger systematic review and meta-analysis assessing the use of Trastuzumab in treating BC during pregnancy included 22 studies involving 22 pregnant women and 23 fetuses. The mean duration of usage averaged 17 weeks, while the average gestational week of delivery was approximately 34.3 weeks. Complications arose in 77.27% of patients during pregnancy, and 56.52% of newborns experienced complications. The primary complication during pregnancy was anhydramnios (68.18%), whereas the primary birth complications included respiratory distress or tachypnea (30%). Following an average follow-up period of 25.28 months, 17.39% (4/23) of the children succumbed to various causes. Notably, patients treated with Trastuzumab during early pregnancy experienced no complications during pregnancy or childbirth (*P* = 0.043). Furthermore, patients over the age of 30 who received Trastuzumab during pregnancy had a higher likelihood of neonatal complications (odds ratio = 7.778, 95% confidence interval = 1.2-50.424, *P* = 0.04) [9].

Trastuzumab with Pertuzumab can also cause serious complications if received during pregnancy in terms of fetal growth retardation and anhydramnios (Table 3) [10]. It has been strongly recommended to use effective contraception methods to prevent pregnancy while on monoclonal antibodies such as Trastuzumab and Pertuzumab [10].

In our practice, we strongly encourage and counsel the use of effective methods of contraception (ideally copper IUD) to prevent pregnancy in premenopausal women with cancer who are receiving systemic therapy to avoid serious complications and treatment interruption, especially in metastatic settings [11]. If a woman becomes pregnant while undergoing anticancer systemic therapy, it is important to co-consult with a medical oncologist and a maternal-fetal medicine specialist to discuss the potential risks and benefits of continuing or stopping treatment. Factors such as the type and stage of cancer, the specific anticancer therapy being used, the timing of the pregnancy, and the overall health of the mother should all be taken into consideration. In some cases, systemic therapy may need to be postponed or modified to minimize the harm to the developing fetus. In other cases, it may be recommended to terminate the pregnancy to prioritize the mother's health and treatment. Each situation is unique, and decisions should be made on an individual basis. Psychiatric and clinical psychology counseling is an important aspect that should not be overlooked for the well-being of both parents. 

The patient received counseling regarding the recommencement of her therapy options, which included a choice between Trastuzumab-Pertuzumab combination therapy or single-agent Trastuzumab. The patient opted to defer her decision for three months as she expressed a desire to continue breastfeeding her child. Currently, there are no defined criteria for termination of Trastuzumab treatment in selected patient groups who have no evidence of disease. Some recommend stopping the treatment after five years. In a single-center retrospective study, 80 patients were involved in the study. The patients were treated with Trastuzumab for a median of 62 (range 12-191) months. A complete response was observed in 60 (75%) patients. The median duration for the development of a complete response was found to be 14.8 months. After discontinuation of Trastuzumab, with a median follow-up period of 32 (range 11-66) months, recurrence occurred in two (13.3%) patients. The study analysis revealed that menopausal status, the number of metastatic sites, and the incorporation of endocrine therapy alongside Trastuzumab were predictive factors for achieving a complete response in HER-2-positive metastatic BC patients undergoing long-term Trastuzumab-based therapy. The study findings suggest that complete remission is attainable in HER-2-positive metastatic BC patients through the use of Trastuzumab-based treatment. Our patient had a favorable prognosis during the pregnancy, given her status with no evidence of disease for at least 3.5 to 4 years before conception.

The interruption of hormonal therapy in the adjuvant setting for early BC has gained momentum in recent years, especially with the recent publication of the POSITIVE trial. This trial concluded the feasibility and safety of a temporary interruption of endocrine therapy for attempting pregnancy among select women with previous early hormone receptor-positive BC [12]. Yet, studies about the feasibility and safety of pregnancy in stage IV BC are still unknown with limited data.

Despite experiencing recurrent cardiac dysfunction during anti-HER-2 therapy over the years, the patient did not encounter any complications during pregnancy. Her cardiac function returned to normal after delivery, consistent with the well-known reversible dysfunction associated with anti-HER-2 therapy [13].

**Table 3:** Potential anomalies related to the intrauterine exposure to Trastuzumab and/or Pertuzumab. RDS, respiratory distress syndrome

Anomaly	Description
Premature birth	Trastuzumab-Pertuzumab can increase the risk of preterm birth, which may lead to various health problems for the newborn.
Growth restriction [10]	Pertuzumab and Herceptin may affect the growth and development of the fetus, leading to intrauterine growth restriction (IUGR), where the baby does not grow at a normal rate.
Low birth weight [10]	Babies exposed to Trastuzumab-Pertuzumab in utero may be more likely to have a lower birth weight, which can have various health implications.
Developmental delays [10]	There might be a risk of developmental delays or cognitive impairments in children exposed to these drugs during pregnancy.
Neonatal RDS [14]	Babies born prematurely due to exposure to Trastuzumab (RDS with Pertuzumab was not reported) may be at an increased risk of developing RDS, a condition where the newborn's lungs are not fully developed.
Cardiac abnormalities [15]	Pertuzumab and Trastuzumab can affect the developing heart of the fetus, potentially leading to cardiac abnormalities.
Renal agenesis [10,16]	Trastuzumab-Pertuzumab can impact the fetal renal system and growth.

## Conclusions

In this report, we present a unique and first reported case in the literature of a woman with metastatic BC who became pregnant while undergoing active anticancer treatment for her condition at our institution. As far as we are aware, this represents the first documented instance of a successful pregnancy and delivery in a stage IV cancer of any type while the patient is receiving active anticancer therapy. The case is unique, and despite the favorable outcome for both the mother and the child, we still do not recommend such an approach for a patient with advanced cancer, given the significant risk of progression and death for the mother and the fetus. A multidisciplinary approach for such a complex case is a must to evaluate the mother’s medical condition by an experienced oncology team along with a maternal-fetal team with support from a psychiatric and psychological evaluation for the mother. The use of effective contraception during anticancer therapy is essential to avoid such a scenario. More research is needed to evaluate the long-term exposure of anti-HER-2 therapies to children, as the follow-up in current studies is limited.
